# Predictors of treatment control in severe chronic urticaria treated with omalizumab

**DOI:** 10.5415/apallergy.0000000000000162

**Published:** 2025-01-08

**Authors:** Miguel Proença, João Marcelino, João Vieira, Joana Guimarães, Carlota Dias, Elza Tomaz

**Affiliations:** 1 Immunology and Allergy Department, Unidade Local de Saúde Arrábida, Portugal; 2 Charité – Universitätsmedizin Berlin, Germany

**Keywords:** Autoimmune, biomarkers, chronic, omalizumab, urticaria

## Abstract

**Background:**

Real-life clinical research on biomarkers that predict therapy outcomes of severe chronic spontaneous urticaria patients receiving omalizumab (OMA) therapy is still limited. For this reason, we aimed to identify biomarkers that predict the response to OMA treatment.

**Methods:**

A single-center, observational, retrospective review of patients with severe chronic urticaria treated with OMA from January 2015 to January 2023 in a Portuguese Immunology and Allergy Department. One-way ANOVA and linear regression were used.

**Results:**

Data on 56 OMA-treated chronic spontaneous urticaria patients shows patients can be divided into 3 groups according to their response to OMA. The first group of 26 patients (46.4%) successfully reduced their IMA dose without experiencing any rebound effects. The second group of 19 (33.9%) patients achieved disease control but could not tolerate a progressive dose reduction, and the third group of 11 (19.6%) patients, required a higher dose to achieve disease control. In group 1, patient age and a favorable clinical response had a positive correlation (*P* = 0.008). The patient’s age was also correlated to the time interval until a dose reduction was tolerated (*r* = 0.69; *P* = 0.005). There was also a negative correlation between the ratio: thyroid peroxidase antibodies/total-IgE and a favorable clinical response (*r* = −0.74; *P* = 0.021). In group 2, thyroid peroxidase antibodies were negatively correlated with a favorable clinical response (*r* = −0.55; *P* = 0.027). In group 3, anti-double-stranded DNA was negatively correlated with a favorable clinical response (*r* = −0,97; *P* = 0,007).

**Conclusion:**

Our study suggests that older patients experience higher success rates with OMA compared to younger individuals, but increasing age is also associated with a longer interval before achieving successful dose reduction. Potential markers of resistance to OMA identified in our cohort included elevated levels of IgG-antithyroid peroxidase antibodies, positive anti-dsDNA antibodies, and a higher IgG-antithyroid peroxidase/total-IgE ratio.

## 1. Introduction

Chronic urticaria (CU) is characterized by the occurrence of wheals, angioedema, or both for more than 6 weeks, presenting either as chronic spontaneous urticaria (CSU) or chronic inducible urticaria (CIndU), depending on the presence of specific triggers [1, 2]. CSU occurs without an identifiable trigger, whereas CIndU manifests in response to physical or environmental stimuli such as pressure, temperature, or exercise [1, 3]. Some patients present with overlapping forms, known as mixed CU, combining features of both spontaneous and inducible urticaria [2].

Epidemiological data suggests that 52% of patients achieve remission within 3 months, and up to 80% within a year [4]. However, 11% of patients continue to experience symptoms beyond 5 years, especially in severe cases, underscoring the challenge in predicting disease duration and outcomes [5]. The substantial and unpredictable impact of CU on quality of life necessitates the use of standardized assessment tools, including the urticaria activity score (UAS7) [6], angioedema activity score [7], CU quality of life (CU-QoL) questionnaire [8], angioedema QoL questionnaire [7], urticaria control test (UCT) [9], and angioedema control test [10]. These tools are crucial in both clinical practice and research for quantifying disease activity, control, and patient burden.

Current guidelines recommend a stepwise treatment approach to CU, beginning with second-generation nonsedating antihistamines (sgAH) as first-line therapy, which can be increased up to 4-fold if necessary based on disease control [1]. Omalizumab (OMA), a recombinant humanized anti-IgE antibody, has been approved since 2014 for patients unresponsive to antihistamines and has shown high efficacy and safety. However, approximately 30% of patients do not achieve symptom control with the standard OMA dose of 300 mg every 4 weeks, highlighting the need for further therapeutic options and predictors of response [11-16].

Identifying clinical and/or biomarkers predictive of treatment response remains an area of active research. Baseline total-IgE levels have been linked to OMA efficacy, with lower levels observed in nonresponders and higher levels predicting a better response [17-19]. Additional proposed markers include C-reactive protein (CRP), which correlates with disease activity and severity, and D-dimers, which are elevated in severe cases and associated with recurrent angioedema [5, 19-21]. Positive autologous serum skin tests and specific autoantibodies such as antithyroid peroxidase (anti-TPO) and anti-dsDNA have been linked to prolonged disease duration and reduced response to OMA, further complicating treatment strategies [20, 22, 23].

Autoimmune markers, including antinuclear antibodies (ANA) and inflammatory cytokines, have been studied as potential indicators of disease severity and resistance to treatment. Although the clinical utility of these markers varies, their presence can help guide clinical decisions and set realistic expectations for patients regarding the severity and duration of their disease [24, 25].

Given the variability in OMA response, this study aims to explore the clinical and laboratory biomarkers associated with 3 critical treatment outcomes: (1) the need for OMA dose increase, (2) tolerance to dose reduction, and (3) relapse after OMA discontinuation. These findings will be based on a cohort of severe CU patients treated in a Portuguese Immunology and Allergy Department, providing real-world insights into factors that influence OMA effectiveness.

## 2. Methods

Ethical approval for this study (Ethical Committee Nrº 011P/VE/INV/2024) was provided by the Ethical Committee NAC of Unidade Local De Saúde Da Arrábida, Setúbal, Portugal (Chairperson Dra. Quitéria Rato) on 26 September 2024.

This study was a single-center, observational, retrospective review conducted at a Portuguese Immunology and Allergy Department, examining patients with severe CU treated with OMA between January 2015 and January 2023. Data collection focused on demographic, clinical, and analytical variables. Clinical data included (1) the duration of CU, (2) the duration, dose, and frequency of OMA treatment, (3) the presence of atopy (defined according to the World Allergy Organization) [26], and (4) treatment schedules.

Patient-reported outcomes were utilized to evaluate disease activity, impact, and control. These included the Weekly UAS7 [6], the UCT [9], the Dermatology Life Quality Index (DLQI) [27], and the CU-QoL Questionnaire [8]. Analytical data encompassed a differential blood count, CRP, erythrocyte sedimentation rate, D-dimers, total serum IgE, Helicobacter pylori screening, ANAs, anti-double-stranded DNA antibodies, and antithyroid antibodies (antithyroid peroxidase antibodies and antithyroglobulin antibodies).

**Figure 1.**
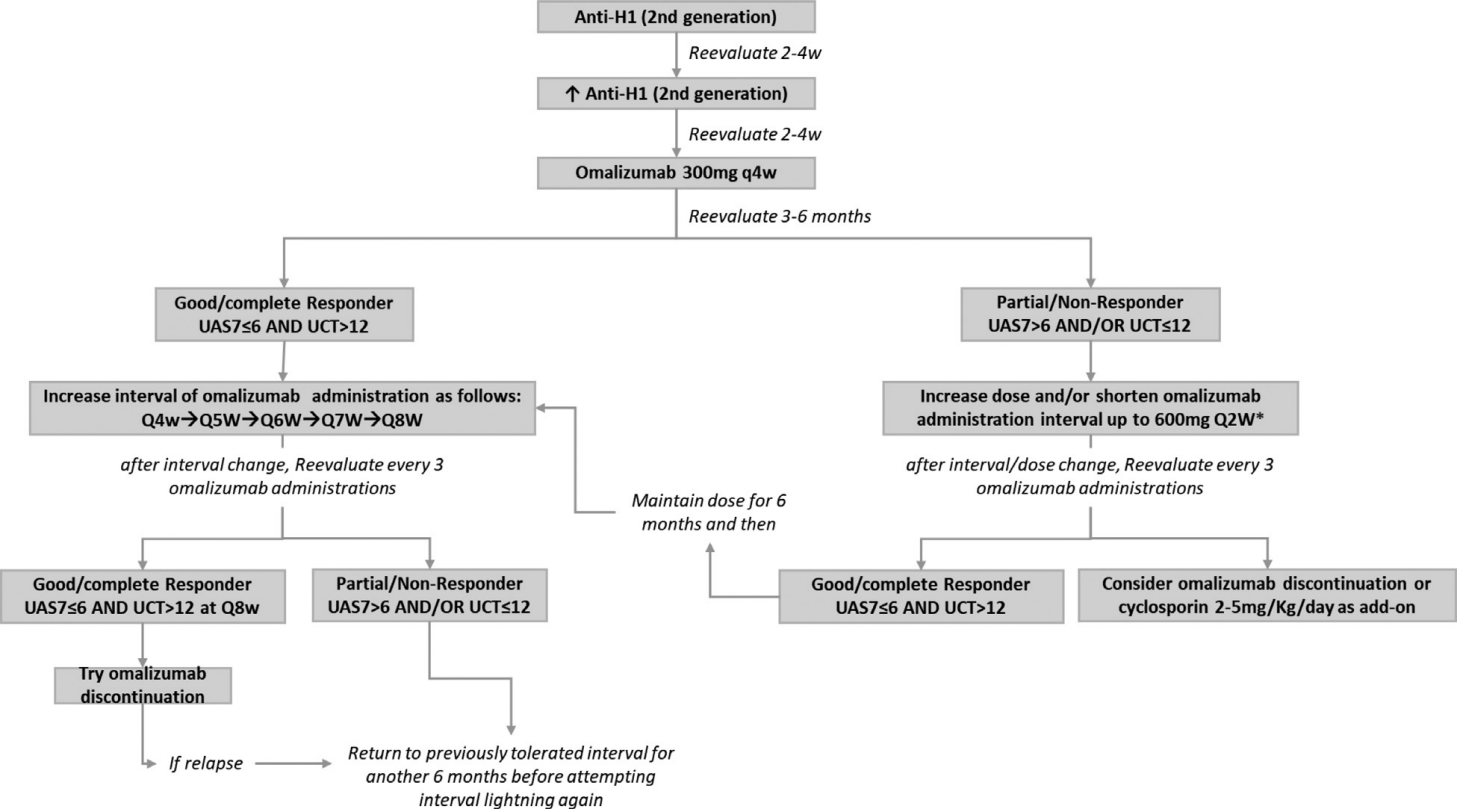
Dose increase to 450 mg and 600 mg is preferred if UAS7 is uniformly elevated during the interval between omalizumab administrations. Shortening of interval is preferred if UAS7 is ≤6 during the first weeks after omalizumab administration, and then worsens. Both strategies can be used until the maximum dose of 600 mg Q2W. UAS7, urticaria activity score; UCT, urticaria control test.

Patients’ responses to OMA were classified using the criteria proposed by Giménez Arnout et al [28], categorizing them into 3 groups: responders with UAS7 scores of 6 or below, partial responders with UAS7 scores between 7 and 15, and nonresponders with UAS7 scores above 15. OMA doses and administration intervals were adjusted according to patient responses (**Fig. 1.**)

All patients began treatment with the standard OMA dose of 300 mg administered subcutaneously every 4 weeks. Spertino et al [14] described a similar approach for OMA dose and/or interval adjustment [29]. In cases where patients were classified as nonresponders or partial responders after 6 months, the dose was escalated to 450 mg, and then, if necessary, to 600 mg every 4 weeks. Conversely, responders were eligible for dose reduction, achieved by extending the administration interval by 1 week every 3 sessions. Dose reduction continued progressively until patients could tolerate an interval of up to 8 weeks, after which OMA cessation was attempted. A reduction in 2nd sgAH was only initiated in those who responded positively to OMA, while partial and nonresponders continued with their initial antihistamine regimen.

Patients were ultimately divided into 3 distinct groups based on their OMA response profile: responders who tolerated dose reduction, responders who did not tolerate dose reduction, and poor responders to the standard OMA dose. Age differences between these groups were assessed using one-way ANOVA, while predictors for the final UCT score and the time until the first dose reduction were identified through linear regression analysis. All statistical analyses were performed using Statistical Package for Social Science version 27.

## 3. Results

A total of 56 patients were included in this study, with a mean age of 41.6 ± 16.4 years (12–74 years). Forty (71.4%) were women. The standard OMA dose was effective in 41 patients (73.2%), while the dose was increased in 12 patients (21.4%) to improve effectiveness. No patients in our cohort were nonresponders to OMA. The baseline demographic and clinical characteristics of the study population are presented in Table 1.

**Table 1.** Clinical characteristics of patients with chronic urticaria

Characteristic	n (%)
Total number of patients	56 (100%)
Age, median (years)	41.6 ± 16.4
Gender, female/male	36/20 (64%/36%)
UAS7 mean value^*^	15.1 ± 9.0
UCT mean value^*^	6.2 ± 3.7
Atopy	28 (50%)
Angioedema	22 (39%)
Chronic spontaneous urticaria (CSU)	**34 (61%**)
Chronic mixed urticaria:	**12 (21%**)
CSU with dermographic urticaria	5 (9%)
CSU with cholinergic urticaria	4 (7%)
CSU with delayed pressure urticaria	3 (5%)
Chronic inducible urticaria (CIndU):	**10 (18%**)
Cold urticaria	4 (7%)
Cholinergic urticaria	3 (5%)
Delayed pressure urticaria	2 (4%)
Heat urticaria	1 (2%)
Concomitant autoimmune disease^†^	16 (28.5%)
Urticaria exacerbation by NSAID	8 (14%)

NSAID, non-steroidal anti-inflammatory drugs; UAS7, urticaria activity score; UCT, urticaria control test.

* Under quadruple dose of 2nd sgAH.

† Thyroid autoimmune disease was the most prevalent n = 14, followed by Discoid Lupus Erythematosus (DLE) n = 1 and Vitiligo n = 1.

Of the patients, 38 (67.8%) had CSU, 10 (17.8%) had mixed CU, including 6 with CSU and symptomatic dermographism, 2 with CSU and cholinergic urticaria, 1 with CSU, symptomatic dermographism and cholinergic urticaria, and 1 with delayed pressure urticaria. Eight patients (14.2%) had isolated CIndU (4 with cholinergic urticaria, 2 with cold urticaria, 1 with heat urticaria, and 1 with delayed pressure urticaria).

At the start of OMA treatment, patients had an average baseline UCT score of 6.2 ± 3.7 and a UAS7 score of 15.1 ± 9.0.

Before starting OMA, patients presented the following laboratory results: (1) a mean total serum IgE of 251.8 ± 234.1 kU/L; (2) a mean eosinophil count of 332.82 ± 624.9 cells/mm³ and a basophil count of 95.49 ± 271.2 cells/mm³; (3) mean D-dimer levels of 624.08 ± 770.9 mcg/mL; (4) positive anti-dsDNA autoantibodies in 6 patients; (5) positive ANAs in 7 patients, with 3 having positivity at dilutions higher than 1:160; (6) positive anti-TPO autoantibodies in 10 patients, with an average level of 52.9 ± 136.8 U/mL; and (7) an IgG-anti-TPO/total-IgE average ratio of 0.80 ± 2.35; The average time until the first OMA dose reduction was 72.3 ± 34.5 weeks. OMA was discontinued in 4 patients (9.8%). Of these, 2 tolerated the discontinuation, while the other 2 experienced mild relapses that were effectively managed with 2nd sgAH, without the need to restart OMA. At the end of the study, patients had an average UCT score of 12.4 ± 3.9 and a UAS7 score of 6.1 ± 8.4.

Patients were stratified into 3 groups according to their response to OMA: (1) responders who tolerated dose reduction, (2) responders who did not tolerate further dose reduction, and (3) poor responders to the standard OMA dose. Clinical and biochemical characteristics of patients stratified by OMA response are presented in Table 2.

**Table 2.** Clinical and biochemical characteristics of patients stratified by omalizumab response to the standard dose (300 mg q4w)

Characteristic	Responders (n = 26)	Partial responders (n = 19)	Poor responders (n = 11)
Age (years)	45.6 ± 15.7	44.7 ± 13.8	31.2 ± 17.7
Female, n (%)	19 (73.1%)	16 (84.2%)	8 (72.7%)
Atopy	7 (25.0%)	13 (46.4%)	8 (28.5%)
Angioedema	5 (22.7%)	7 (31.8%)	10 (45.4%)
Autoimmune disease	4 (28.5%)	5 (35.7%)	7^†^ (50.0%)
Baseline UCT score^*^	6.4 ± 3.5	6.2 ± 3.9	6.0 ± 3.7
Baseline UAS7 score^*^	15.0 ± 8.9	15.2 ± 9.1	15.1 ± 9.0
Total serum IgE (kU/L)	312.7 ± 277.8	200.0 ± 197.0	205.3 ± 168.9
Eosinophils (/mm³)	439.4 ± 845.3	160.0 ± 183.9	408.6 ± 611.7
Basophils (/mm³)	94.7 ± 277.9	39.0 ± 101.2	178.2 ± 405.4
D-dimer level (mcg/mL)	548.4 ± 905.4	612.8 ± 732.9	798.0 ± 678.3
Positive anti-dsDNA, n (%)	0 (0%)	1 (5.3%)	5 (45.5%)
Positive ANA, n (%)	1 (3.8%)	2 (10.5%)	4 (36.4%)
Anti-TPO (U/mL)	39.9 ± 141.8	53.8 ± 124.7	70.2 ± 163.3
IgG-anti-TPO/total-IgE ratio	0.48 ± 1.65	1.15 ± 3.18	0.59 ± 1.2
Omalizumab dose reduction (weeks)	49.5 ± 16.6	75.0 ± 12.2	111.5 ± 49.3
UCT score at study end	13.8 ± 3.0	11.8 ± 3.7	10.9 ± 5.0
UAS7 score at study end	2.9 ± 4.6	5.1 ± 7.9	10.9 ± 13.7
Omalizumab discontinuation, n (%)	4 (15.4%)	0 (0%)	0 (0%)
Relapse after discontinuation, n (%)	2 (7.7%)^‡^	-	-
Potential biomarkers predicting UCT >12	Anti-TPO/IgE ratio	Anti-TPO	Anti-dsDNA

Responders (group 1): Older age predicted a longer time until the first dose reduction. Anti-TPO/IgE ratio was a predictor for achieving UCT >12. Partial responders (group 2): had the lowest eosinophil counts and slightly lower serum IgE levels. Anti-TPO was a predictor for UCT >12. Poor responders (group 3): had a significantly younger age and a higher rate of autoimmune markers, with anti-dsDNA predicting lower UCT scores.

ANA, antinuclear antibodies; TPO, thyroid peroxidase; UAS7, urticaria activity score; UCT, urticaria control test.

To the standard dose (300 mg q4w).

* Score while taking quadruple dose of 2nd sgAH.

† DLE and Vitiligo patients included.

‡ these two patients experienced mild relapses manageable with 2nd nsAH.

In the first group, OMA dose reduction was tolerated without rebound effects. This group included 26 patients (46.4%) with a mean age of 45.6 ± 15.7 years (range: 15–74), of whom 19 (73.1%) were female. At the start of OMA treatment, these patients had: (1) a mean total serum IgE of 312.7 ± 277.8 kU/L; (2) 439.4 ± 845.3 eosinophils/mm³ and 94.7 ± 277.9 basophils/mm³; (3) D-dimer levels of 548.4 ± 905.4 mcg/mL; (4) negative anti-dsDNA autoantibodies in all patients; (5) positive ANAs in 1 patient; (6) positive anti-TPO autoantibodies in 6 patients, with an average level of 39.9 ± 141.8 U/mL; and (7) an IgG-anti-TPO/total-IgE ratio of 0.48 ± 1.65. The average time to the first dose reduction was 49.5 ± 16.6 weeks. OMA was discontinued in 4 patients (9.8%), of whom 2 tolerated the discontinuation and 2 experienced mild relapses, as mentioned above. At the end of the study, this group had an average UCT score of 13.8 ± 3.0 and a UAS7 score of 2.9 ± 4.6.

The second group included 19 patients (33.9%) with a mean age of 44.7 ± 13.8 years (range: 13–67), 16 (84.2%) of whom were female. At the start of OMA treatment, these patients presented: (1) a mean total serum IgE of 200.0 ± 197 kU/L; (2) 160 ± 183.9 eosinophils/mm³ and 39 ± 101.2 basophils/mm³; (3) D-dimer levels of 612.8 ± 732.9 mcg/mL; (4) positive anti-dsDNA antibodies in 1 patient; (5) positive ANAs in 2 patients; (6) positive anti-TPO antibodies with a mean of 53.8 ± 124.68 U/mL; and (7) an IgG-anti-TPO/total-IgE ratio of 1.15 ± 3.18. The average time to the first dose reduction was 75.0 ± 12.2 weeks, and OMA was not discontinued in any patients. At the end of this study, these patients averaged a UCT score of 11.8 ± 3.7 and a UAS7 score of 5.1 ± 7.9.

In the third group, which included patients whose OMA dose was increased to achieve disease control, there were 11 patients (19.6%) with a mean age of 31.17 ± 17.7 years (range: 12–59), and 8 (72.7%) were female. At the start of OMA treatment, these patients presented: (1) a mean total serum IgE of 205.3 ± 168.9 kU/L; (2) 408.6 ± 611.7 eosinophils/mm³ and 178.2 ± 405.4 basophils/mm³; (3) D-dimer levels of 798.0 ± 678.3 mcg/mL; (4) positive anti-dsDNA antibodies in 5 patients; (5) positive ANAs in 4 patients; (6) anti-TPO antibodies with a mean of 70.2 ± 163.3 U/mL; and (7) an IgG-anti-TPO/total-IgE ratio of 0.59 ± 1.2. The average time to the first dose reduction was 111.5 ± 49.3 weeks. At the study’s conclusion, patients in this group averaged a UCT score of 10.9 ± 5.0 and a UAS7 score of 10.9 ± 13.7.

In this group, a thorough differential diagnosis was performed, including an evaluation for urticarial vasculitis and Schnitzler syndrome. Notably, there was no evidence of any systemic symptoms, no specific laboratory test, or hypocomplementemia (reduced levels of C3, C4, and/or C1q). This absence of hypocomplementemia and systemic manifestations suggests that these cases were not consistent with urticarial vasculitis, which could confound the response to treatment.

**Figure 2.**
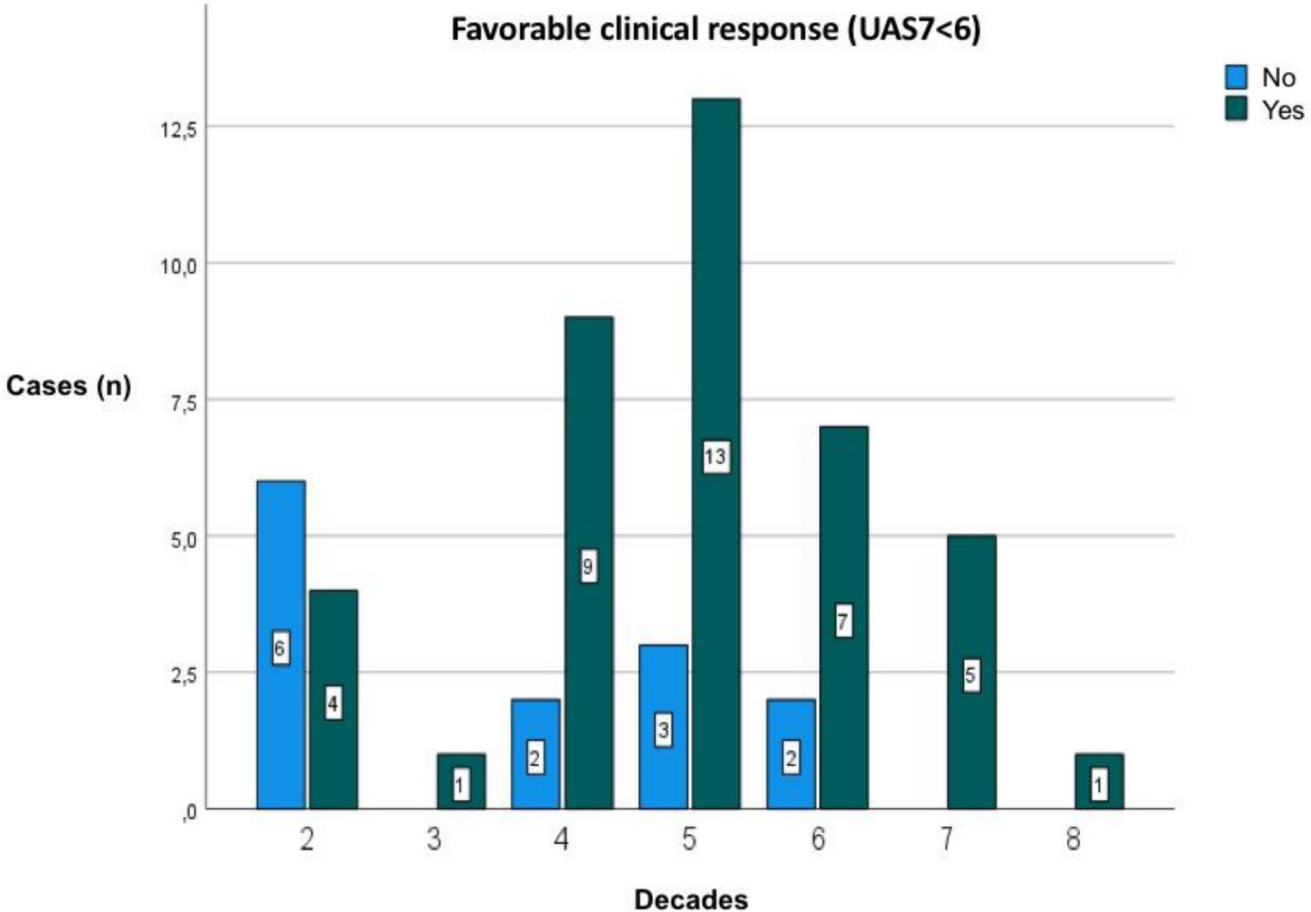
A favorable clinical response according to the patient’s age. Older individuals had a positive response to omalizumab therapy, whereas younger patients had a partial and/or negative response to standard therapy (*P* = 0.028). UAS7, urticaria activity score.

**Figure 3.**
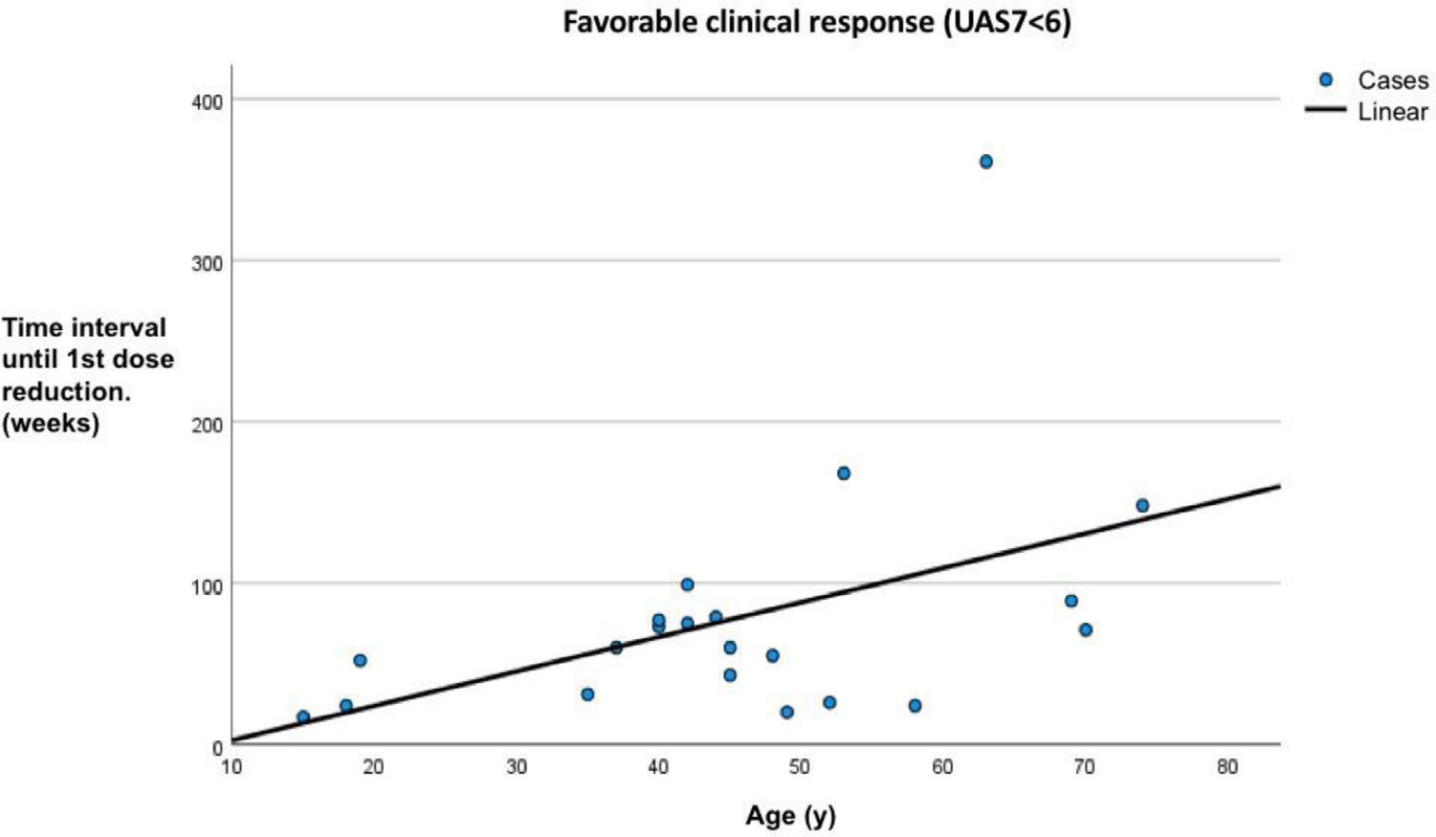
A time interval until 1st dose reduction according to the patient’s age. Those above the age of 50 responded to omalizumab far more gradually (*r* = 0.69; *P* = 0.005). UAS7, urticaria activity score.

Comparing the groups, patients in the third group (nonresponders to the standard OMA dose) were significantly younger than those in the other groups (*P* = 0.028). The relationship between OMA response and patient age is illustrated in Figure 2. In the first group, age was a predictor of the time until the first dose reduction, with older patients taking longer to reach dose reduction (*r* = 0.69; *P* = 0.005), as shown in Figure 3. Biomarkers predicting a final UCT >12 were anti-TPO/IgE ratio in the first group (*r* = −0.74; *P* = 0.021), anti-TPO in the second group (*r* = −0.55; *P* = 0.027), and anti-dsDNA in the third group (*r* = −0.97; *P* = 0.007).

The total serum IgE was higher in the first group (312.7 ± 277.8 kU/L) compared to the third group (205.3 ± 168.9 kU/L), although this difference was not statistically significant. The eosinophil count was similar between the first group (439.4 ± 845.3/mm³) and the third group (408.6 ± 611.7/mm³), but lower in the second group (160 ± 183.9/mm³), with no significant differences. The third group had a higher overall rate of autoimmune diseases, 63.6% (7 out of 11 patients), including 5 cases of thyroid autoimmune disease, 1 case of discoid lupus erythematosus, and 1 case of vitiligo; however, there was no correlation with the final UCT score.

## 4. Discussion

The management of severe CU with OMA has proven effective for many patients; however, individual responses vary significantly, demanding personalized treatment approaches. Identifying clinical and biochemical characteristics that predict response, need for dose escalation, and tolerance to dose reduction remains a critical challenge in optimizing therapy. In our cohort, we explored the association of demographic, clinical, and laboratory parameters with treatment outcomes, highlighting distinct patterns among responders, poor responders, and those attempting OMA discontinuation. These characteristics are summarized in Table 3.

**Table 3.** Clinical and biochemical predictors of omalizumab updosing, dose reduction tolerance, and relapse in chronic urticaria patients

Patterns of response	Potencial clinical and biochemical predictors
Need for omalizumab updosing (poor responders)	Younger patients were more likely to require dose escalation to achieve disease control.
	Higher prevalence of autoimmune markers, such as anti-dsDNA and ANA, correlated with lower UCT scores.
	Angioedema was significantly more prevalent in the poor responder group (45.5%) compared to responders (22.7%) and those tolerating dose reduction (31.8%).
	Anti-dsDNA titers negatively correlated with final UCT (*r* = −0.97; *P* = 0.007), indicating poor disease control even at higher doses.
Tolerance of dose reduction (responders)	Older age was associated with a longer time to tolerate dose reduction, indicating a slower but sustained response (*r* = 0.69; *P* = 0.005).
	Elevated total serum IgE levels were linked to better tolerance and treatment success (UCT >12).
	Lower anti-TPO/IgE ratio correlated with improved disease control (*r* = −0.74; *P* = 0.021).
Relapse after omalizumab discontinuation	A small subset of responders attempted discontinuation, with mixed outcomes.
	No specific biomarkers identified to predict relapse, emphasizing the need for cautious discontinuation and close monitoring.

ANA, antinuclear antibodies; TPO, thyroid peroxidase; UCT, urticaria control test.

### 4.1. Need for omalizumab updosing (poor responders)

While many studies have established pretreatment biomarkers to predict OMA success in CU, few have examined the correlation between age and therapeutic effectiveness [11, 12, 30, 31]. In our study, we observed a positive correlation (*P* = 0.028) between patient age and a favorable clinical response to OMA as shown in Figure 2. Younger patients were more likely to require dose escalation to achieve disease control, indicating age as a significant factor in predicting treatment response. To the author’s knowledge, this is the first study identifying this correlation between age and OMA effectiveness, and additional data is needed to confirm this finding.

Anti-dsDNA antibodies can be observed in healthy individuals, as well as in other autoimmune syndromes [32], infections, and cancer [26, 27, 33]. This opens the door to a potential role of anti-dsDNA antibodies in CU real-life studies. Higher levels of autoimmune markers, such as anti-dsDNA and ANA, were more prevalent in poor responders, with anti-dsDNA specifically correlating with lower UCT scores (*r* = −0.97; *P* = 0.007), suggesting poor disease control even at higher doses.

### 4.2. Tolerance of dose reduction (responders)

Interestingly, although a higher percentage of older patients were responsive to OMA at a standard dose, this group was less likely to tolerate a dose reduction. Our data indicates that the higher the age (specifically above 50), the longer it took for patients to successfully tolerate a dose reduction (*r* = 0.69; *P* = 0.005) (Fig. 3). This aligns with previous reports showing that older age is associated with a slower but sustained response to treatment [11, 28].

Elevated total serum IgE levels and the anti-TPO/IgE ratio were identified as significant predictors of treatment tolerance and success, with a lower ratio linked to better control (UCT >12). In our cohort, there was a negative correlation between the ratio of IgG-anti-TPO antibodies to total IgE and the final UCT (*r* = −0.74; *P* = 0.021). This is consistent with findings by Maurer et al [34-36], who reported that a high TPO/low IgE ratio was linked to autoimmune CSU, suggesting its potential use in daily practice to identify patients more likely to be resistant to OMA treatment. Furthermore, the average ratio of IgG-anti-TPO levels to IgE levels in autoimmune CSU patients was approximately 15 times greater than in partial autoimmune CU patients (*P* < 0.0001) and about 35 times greater than in nonautoimmune CSU, as reported by Schoepke et al [25]. In our cohort, individuals who tolerated partial interval lengthening (group 2) had slightly higher pre-OMA TPO antibody levels than those who remained on the standard dose. A negative correlation was observed between pre-OMA TPO antibody levels and the final UCT in this group (*r* = −0.55; *P* = 0.027), reinforcing the role of autoimmune markers in influencing treatment adjustments.

### 4.3. Relapse after omalizumab discontinuation

Relapse after OMA discontinuation was observed in a small subset of responders, with mixed outcomes. Notably, no clear biomarkers predicted relapse, underscoring the need for cautious discontinuation and close monitoring. This finding highlights a gap in identifying predictors for safe treatment cessation, emphasizing the need for future research in this area.

Our results suggest that age plays a significant role in OMA treatment response, with younger patients more likely to require dose escalation and older patients showing slower adaptation to dose reduction. Additionally, specific autoimmune markers, such as anti-dsDNA and the anti-TPO/IgE ratio, were associated with treatment resistance, indicating their potential utility in clinical decision-making. This study has limitations, it is retrospective in design and has a limited sample size, restricting its capacity to generalize the results. Further studies are warranted to validate these findings.

## 5. Conclusion

Our study suggests that patients over 50 years old experience higher success rates with OMA compared to younger individuals, but increasing age is also associated with a longer interval before achieving successful dose reduction. This reflects the importance of carefully considering the timing of dose adjustments in older patients.

Potential markers of resistance to OMA identified in our cohort included elevated levels of IgG-anti-TPO antibodies, positive anti-dsDNA antibodies, and a higher IgG-anti-TPO/total-IgE ratio. These findings highlight the relevance of these biomarkers in predicting treatment response, although their utility should be further validated in larger studies.

This study has limitations, including its retrospective design and relatively small sample size, which may limit the generalizability of the results. Future prospective studies with larger cohorts are needed to confirm these findings and refine the identification of clinical and biochemical predictors of OMA response in CU patients.

## Conflicts of interest

The authors have no financial conflicts of interest.
